# Combined Effects of Non-Conforming Fly Ash and Recycled Masonry Aggregates on Mortar Properties

**DOI:** 10.3390/ma9090729

**Published:** 2016-08-25

**Authors:** Ana Isabel Torres-Gómez, Enrique F. Ledesma, Rocio Otero, José Maria Fernández, José Ramón Jiménez, Jorge de Brito

**Affiliations:** 1 Construction Engineering Area, EPS de BELMEZ, University of Córdoba, Córdoba 14002, Spain; p72togoa@uco.es; 2 Department of Mechanics, EPS de BELMEZ, University of Córdoba, Córdoba 14002, Spain; efledesma@uco.es; 3 Inorganic Chemical Area, Faculty of Science, University of Córdoba, Córdoba 14071, Spain; b42otizr@uco.es; 4 Inorganic Chemical Area, EPS de BELMEZ, University of Córdoba, Córdoba 14002, Spain; 5 CERIS-ICIST, Instituto Superior Técnico, University of Lisbon, Av. Rovisco Pais, Lisbon 1049-001, Portugal; jb@civil.ist.utl.pt

**Keywords:** mortars, non-conforming fly ash, powdered addition, recycled aggregates, construction and demolition waste

## Abstract

This work evaluates the effects of using non-conforming fly ash (Nc-FA) generated in a thermoelectric power plant as filler material for mortars made with natural sand (NA) and recycled sand from masonry waste (FRMA). The incorporation of powdered recycled masonry filler (R-MF) is also tested as an alternative to siliceous filler (Si-F). Three families of mortars were designed to study: the effect of replacing Si-F with Nc-FA on mortars made with NA; the effect of replacing Si-F with Nc-FA on mortars made with 50% of NA and 50% of FRMA; and the effect of replacing Si-F with R-MF on mortars made with NA and FRMA. Replacing Si-F with Nc-FA is a viable alternative that increases the mechanical strength, the workability and durability properties and decreases the shrinkage. The use of FRMA and Nc-FA improved the mechanical strength of mortars, and it slightly increased the shrinkage. The replacement of Si-F with R-MF on mortars made with FRMA is not a good alternative, because it has a negative impact on all of the properties tested. This work can help both to reduce cement and natural resources’ consumption and to increase the recycling rate of Nc-FA and FRMA.

## 1. Introduction

Coal is still a major fuel for energy production in Europe (EU-28). According to the Eurostat website [1], the member states of EU-28 consumed 308 million tonnes (MMt) of hard coal and 432 MMt of lignite in 2013. Pulverized coal is burned in thermoelectric power plants, where a big quantity of coal combustion products (CCPs) is generated. Depending on the coal’s mineral components and the combustion technique, six different CCPs can be identified: fly ash (FA), bottom ash (BA), boiler slag (BS), fluidized bed combustion ash (FBC), semi-dry absorption product (SDA) and flue gas desulphurization gypsum (FGD). According to the European Coal Combustion Products Association (ECOBA) [2], approximately 105 MMt of coal combustion products (CCPs) will be produced in Europe (EU-28) in 2015. Fly ash is the most important CCP because it accounts for nearly 68% of the total amount [3].

In Spain, there are 22 thermoelectric power plants that produce 10 MMt of FA and BA per year, although with a clear downward trend. However, according to the annual reports published by the Spanish electrical companies, approximately 60% of the FA is used as cement raw materials, concrete addition, clay soil stabilization for road construction or filling application, the remaining material being deposited in landfills or stockpiled. These data are comparable with those of other countries, such as the United Kingdom [4].

In accordance with the European Union Decision 2014/955/UE [5], FA from coal combustion is cataloged as waste with code 10 01 02. The European Waste Framework Directive (Directive 2008/98/EC) [6] specifies that any material considered as waste must be recovered and achieve end of waste (EoW) status before it may be used again. Any FA, after a short time of storage, goes directly from the power plant to the end user, ceases to be waste and becomes a secondary raw material. FA is subject to the REACH regulation (Registration, Evaluation, Authorization and Restriction of Chemicals) [7].

**Figure 1:**
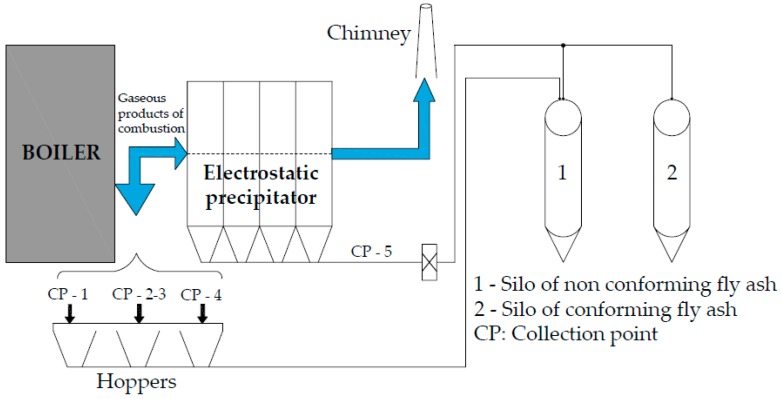
Scheme of fly ash production in a conventional thermoelectric power plant.

The chemical composition and mineralogy of FA is determined by the coal source and the thermoelectric power plant typology. In Spain, most plants are conventional thermoelectric power plants. Figure 1 shows the flowchart of fly ash production in a conventional thermoelectric power plant. FA is contained in combustion gases from furnaces fired with hard coal or lignite at 1100 °C–1400 °C. Five collection points are located across the combustion gas stream from the outlet of the boiler to the chimney: CP-1 (saver), CP-2 (secondary air heater), CP-3 (primary air heater), CP-4 (air pre-heaters) and CP-5 (electrostatic precipitator). Ash collection in points CP-1–CP-4 is performed by gravity sedimentation in hoppers installed at the bottom. The collected ash is conveyed to a silo where it is stored (Silo 1). Before leaving the chimney, the combustion gases are conducted to an electrostatic precipitator (CP-5), where suspended particles are ionized and attracted to the collector plates, a system of hammers hitting the collector plates getting ash to fall to a system of hoppers located at the bottom. This ash is conveyed and stored in a different silo.

Only FA collected from the electrostatic precipitator (CP-5) is used as conforming FA in accordance with the European standard UNE-EN 450-1:2013 [8] and UNE-EN 450-2:2006 [9]. This FA contains a heterogeneous mixture of SiO_2_, Al_2_O_3_ and Fe_2_O_3_ and meets the specifications and conformity criteria to be used as mineral addition in concrete production [10]. Conforming FA is widely demanded by the construction industry, and its production is not a problem for thermoelectric power plants.

The remaining FA collected from CP-1 and CP-4 does not meet the established specifications and conformity criteria required by these standards, especially because it exceeds the fineness specification. If the mass retained by the sieve 0.045 mm is over 40% UNE-EN 933-10:2010 [11], the ash is considered non-conforming FA. This non-conforming FA has been used as cement raw materials by cement companies in Spain. From the beginning of the construction industry crisis in 2008, non-conforming FA is no longer demanded or has great difficulties being placed on the market. For this reason in Spain, most of the non-conforming FA is stockpiled in the plant or deposited in landfills. Non-conforming FA represents between 30% and 40% of the total FA produced, which reveals the extent of the environmental problem.

The stockpiling or depositing in landfills of non-conforming FA is against the waste management hierarchy established by the Waste Framework Directive (Directive 2008/98/EC) [6], according to which disposal is the last and worst option. This justifies the need to study viable alternatives for the use of non-conforming FA. The use of untreated non-conforming FA as filler material in mortar production is an understudied alternative.

Masonry mortar is a mixture of sand, cement and water, mineral addition (filler) and admixtures. Sand is a nonrenewable natural resource extracted from rivers, seashores or rock crushing, and its consumption entails a high environmental impact [12]. One way to reduce natural sand consumption is to use fine recycled aggregates obtained from construction and demolition waste (CDW) as recycled sand [13,14,15,16,17]. CDW is a priority waste stream in the European Union. This waste is mainly composed of concrete and masonry waste (European Commission, Environmental Directorate-General (DG ENV), 2011) [18]. In Mediterranean countries, the main components of masonry waste are ceramic bricks and mortar [13]. The Waste Framework Directive 2008/98/EC [6] establishes a minimum recycling rate of 70% for CDW in 2020. Currently, in Spain, the fine fraction obtained from the recycling process of masonry waste is underutilized, and in most cases, it is deposited in landfill or stored in recycling plants. The use of recycled sand is a viable alternative that would help to increase the recycling rate of CDW.

Ledesma et al. [16] demonstrated that a maximum replacement ratio of up to 50% of natural sand with recycled masonry sand can be achieved to manufacture a type M-10 masonry mortar. For this purpose, a CEM-II/BL 32.5 N in a volumetric proportion of cement-to-aggregate of 1:5 was used. Better results were obtained in a previous work of Jiménez et al. [13], where the same recycled sand and a pozzolanic cement CEM-IV/A (V) 32.5 N were used. These improved results were attributed to the type of cement, whose composition had 29% of conforming FA. Other authors have proven that the combined effect of coal fly ash and recycled concrete aggregates (RCA) improves concrete properties [19]. These results are explained by the pozzolanic reactions between FA and Ca(OH)_2_ contained in RCA. Kou and Poon [20] found long-term improvements by using a replacement ratio of 50% of natural gravel with coarse RCA and a replacement ratio of up to 25% of cement by conforming FA. There are not enough studies about the combined effect of recycled aggregates from masonry waste and fly ash.

Mineral addition used as filler in the manufacture of mortars is obtained by grinding siliceous or limestone rocks. According to the von Rittinger law, the required energy to carry out the grinding of particles is proportional to the square root of particle size, which explains why these materials have much embodied energy. Silva et al. [21] and Braga et al. [22] used ultrafine particles of red clay brick and concrete waste, respectively, in mortar production. The use of untreated non-conforming FA or powdered masonry waste as filler material in mortar production is an understudied alternative.

This work investigates the effects of using non-conforming fly ash (generated in a conventional thermoelectric power plant) as filler material in mortar made with natural and recycled sand from masonry waste. The incorporation of powdered recycled masonry aggregates is also tested as an alternative to natural filler.

The emerging economic recovery in the construction sector in Spain is an opportunity to incorporate this waste in the manufacturing of masonry mortars. From an environmental point of view, the use of stockpiled non-conforming FA and recycled aggregates from masonry waste is a great opportunity to reduce natural sand consumption and promote a higher added value for some by-products currently underutilized in Spain.

## 2. Material and Methods

### 2.1. Mortars Design

To evaluate the combined effect of non-conforming fly ash and recycled aggregates from masonry waste on mortar’s properties, three families of mortar were designed:

- Family-1: dedicated to evaluate the effect of replacing siliceous filler (Si-F) with non-conforming fly ash (Nc-FA) on mortars made with natural sand (NA). Three replacement ratios of Si-F by Nc-FA were tested: 0% (reference mortar), 50% and 100%. A constant dry mass proportion of 1:7:0.6 (cement: natural sand: siliceous filler) was used in the reference mortar;
- Family-2: dedicated to evaluate the effect of replacing Si-F with Nc-FA in mortar made with 50% of NA and 50% of recycled sand from masonry waste (FRMA). NA was replaced with FRMA by volume. This percentage was selected based on the previous works of Ledesma et al. [16] and Fernández-Ledesma et al. [17], both of them concluding that a maximum replacement of up to 50% of natural sand with recycled sand from CDW can be admitted without the hardened mortar properties being affected. Three replacement ratios of Si-F with Nc-FA were tested: 0% (reference mortar), 50% and 100%. At this stage, the effect of FRMA on mortar's properties can be also analyzed;
- Family-3: dedicated to evaluate the effect of replacing Si-F with ultrafine particles of masonry waste (recycled masonry filler: R-MF) on mortars made with 100% NA and 50% of NA and 50% of FRMA. A 100% replacement ratio of Si-F with R-MR was tested.

In all families, the replacement of Si-F by Nc-FA and R-MF was made by mass. Table 1 shows the nomenclature of mixes, the relative proportion of NA/FRMA and the percentage of utilization of Si-F, Nc-FA and R-MF for each mortar.

### 2.2. Material Characterization

Five natural and recycled aggregates were selected:

- Natural siliceous sand taken from the quarry of a river (NA);
- Recycled sand obtained from crushing and screening masonry waste (FRMA). The components according to UNE-EN 933-11:2009 [23] were: ceramics 53.9%; mortar 39.8%; natural aggregates 5.7%; concrete 0.4%; plasters 0.2%. This recycled sand was used in previous works [13,16];
- Siliceous filler (Si-F) obtained by grinding siliceous rock and commercialized by Roig and Lorda SA;
- Ultrafine particles of masonry waste (R-MF). This material was produced in the laboratory by introducing 5.0 kg of FRMA in the “Los Angeles machine” UNE 1097-2:2010. [24] The material was subjected to 3000 revolutions with 23 0.445 g balls;
- Non-conforming FA (Nc-FA) obtained from the combustion of hard coal and anthracite stockpiled in the thermoelectric power plant of Puente Nuevo, located in the north of Córdoba (Spain).

Table 2 shows the particle size distribution UNE-EN 933-1:2012 [25] of the aggregates used in our study. The NA and FRMA have a continuous particle size distribution (0/4 mm) and meet the specifications of the standard UNE-EN-13139:2003 [26]. The FRMA showed a higher uniformity coefficient, which gives a higher compactness [27,28].

Table 3 shows the physical-chemical and mechanical properties of NA and FRMA. Both types of sands had a similar percentage of sand equivalent. FRMA showed a higher content of fines, higher water absorption, lower density and lower resistance to fragmentation. These values are typical of fine recycled aggregates from CDW [29]. Regarding the chemical properties, FRMA showed slightly higher values than those required by UNE-EN 13139:2003 [26] for the following properties: acid soluble sulfates, total sulfur compounds and soluble salts. However, this aggregate has been used in previous studies by the authors with satisfactory results [13,16]. In accordance with UNE-EN 1744-1:2010 [30], no organic compounds that could alter the setting of cement were detected. No alkali-silica and alkali-silicate reactivity according to UNE 146507-1:1999 EX [31] was detected.

**Figure 2:**
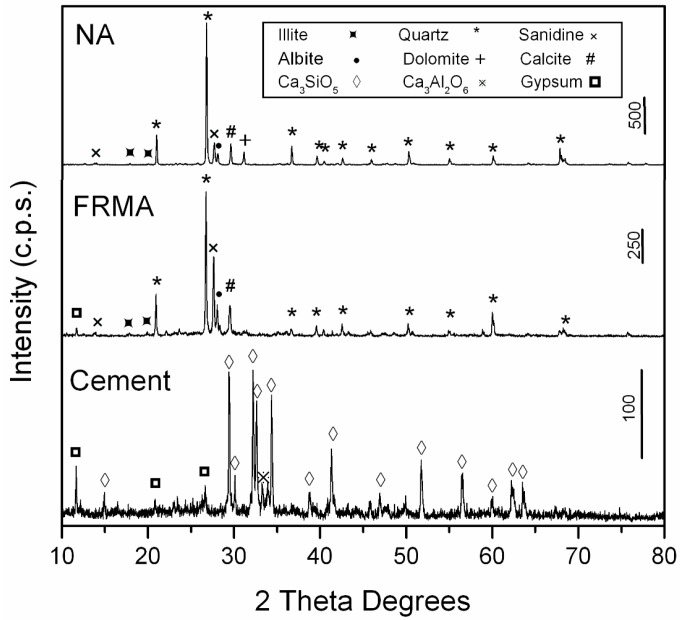
PXRD patterns of NA and FRMA aggregates and cement.

The PXRD patterns of the cement and the aggregates are shown in Figure 2. The main crystalline phase was quartz (33-1161) [35] for both NA and FRNA aggregates, and the degree of presence of calcite (05-0586) [35] was low. The NA sample presented a small amount of dolomite (36-0426) [35], and FRNA showed a small amount of gypsum (42-0551) [35]. The other phases were illite (02-0056) [35], albite (10-0393) [35] and sanidine (10-0357) [35]. The dominant phase in the cement sample was C_3_S (Ca_3_SiO_5_), and there were small amounts of other phases, such as gypsum (33-0311) [35] and Ca_3_Al_2_O_6_ (32-0150) [35].

**Figure 3:**
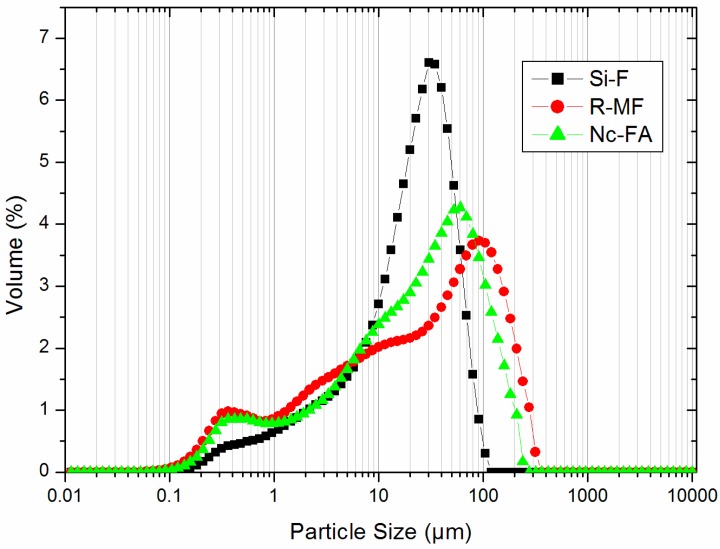
Particle size distribution of Si-F, R-MF and Nc-FA.

In order for the materials to be used as filler, they must meet the following condition: more than 85% by weight must pass through the 0.125-mm sieve and more than 70% through the 0.063-mm sieve UNE-EN-13139:2003 [26]. The Si-F and Nc-FA met both criteria, but the R-MF showed a larger particle size (Table 2). For a more detailed analysis of the particle size distribution in these materials, a Mastersizer-S laser diffraction particle size analyzer equipped with a small volume dispersion unit (Malvern Instruments) was used. Before measuring, the samples were dispersed in ethanol for 10 min. The fillers presented the particle size distribution curves shown in Figure 3. In general, wide distributions in particle size were observed in all samples, although Si-F presented a narrower size distribution than R-MF and Nc-FA. Si-F had a smaller particle size with a maximum distribution of around 30 microns and a very uniform particle size. Nc-FA had a larger particle size with a maximum distribution of around 70 microns. This big amount of particles whose size exceeds 45 microns prevents their use as mineral addition for concrete production in accordance with UNE-EN 450-1:2013 [8] and justifies its nonconformity. R-MF has the largest size with a maximum of around 90 microns. These results are consistent with those shown in Table 2.

Table 4 shows the dry particle density and the bulk density of all filler materials. The Si-F had a higher density of particles, followed by the R-MF and Nc-FA. However, the Nc-FA presented the highest bulk density; this can be explained because its particle size distribution is more continuous than the Si-F (Figure 3), and therefore, it fills the voids between particles better.

**Figure 4:**
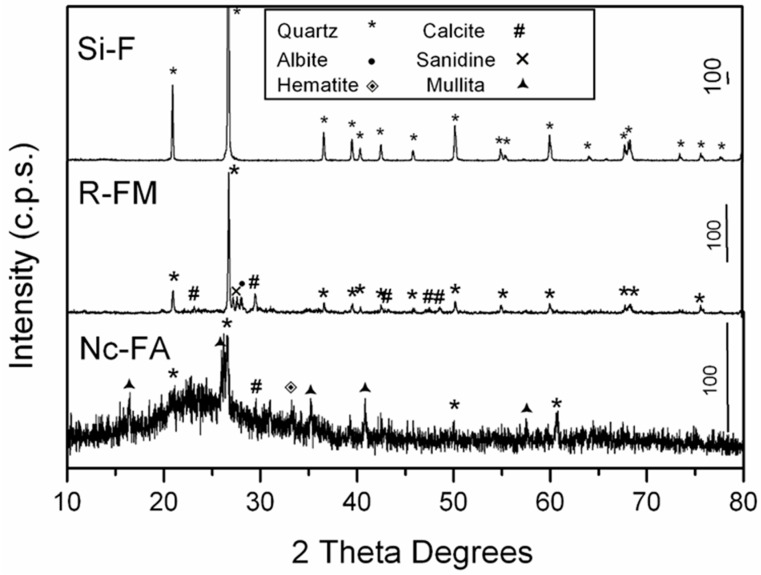
PXRD patterns of Si-F, Nc-FA and R-MF.

**Figure 5:**
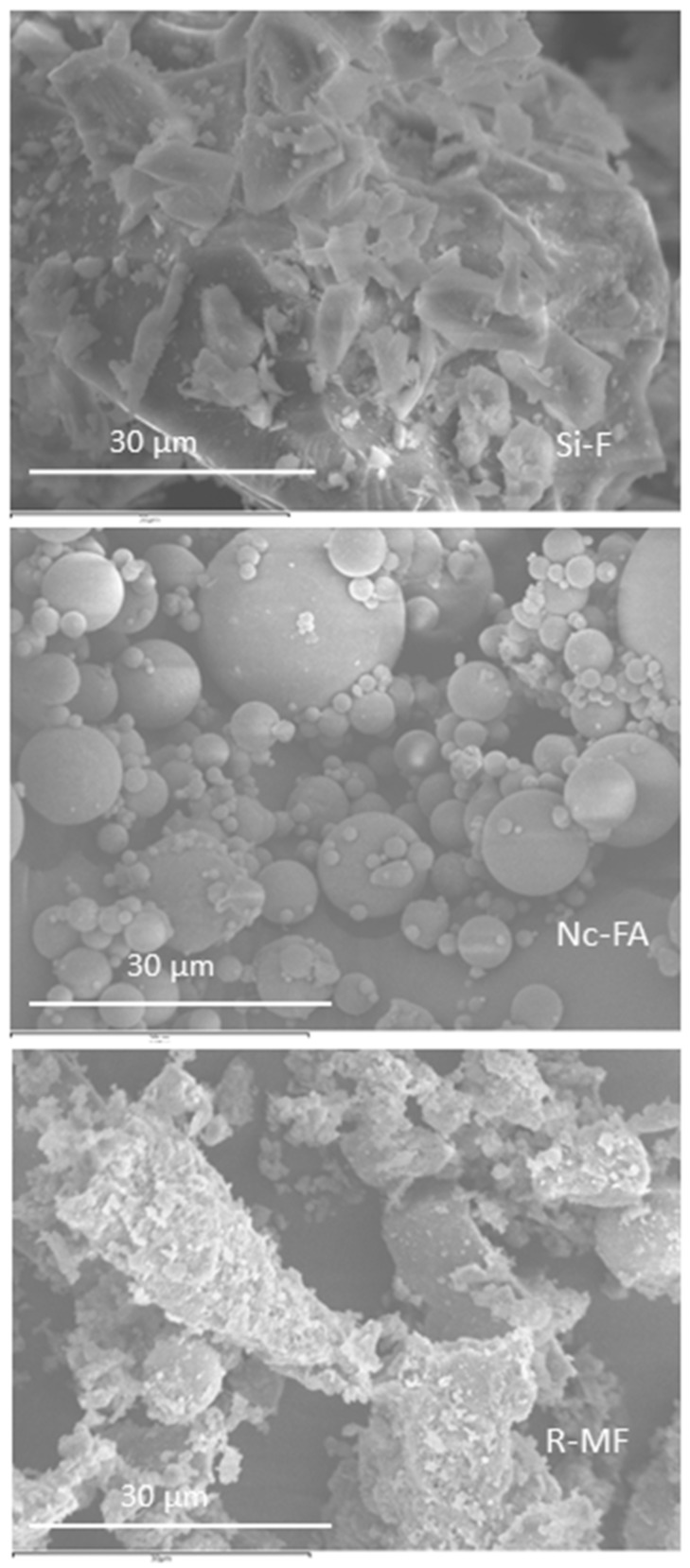
SEM images of Si-F, Nc-FA and R-MF.

The main mineral phases in Si-F, Nc-FA and R-MF were identified using a Siemens D-5000 instrument with Cu Kα radiation. Figure 4 shows the results of the X-ray diffraction patterns (PXRD) of Si-F, Nc-FA and R-MF. The main compound of Si-F was quartz (33-1161) [35], while the R-MF showed quartz (33-1161) [35] and calcite (05-0586) [35] and a small amount of albite (10-0393) [35] and sanidine (10-0337) [35]. The Nc-FA presented quartz (33-1161) [35], mullite (15-0776) [35] and a small amount of calcite (05-0586) [35] and hematite (33-0664) [35]. Quartz (33-1161) [35] and mullite (15-0776) [35] are the major crystalline constituents of low-calcium ash [10]. Fly ash with low calcium chemically reacts with limestone, which is a slow pozzolanic reaction that takes time [10]. The particle shape was analyzed using a scanning electron microscope. Figure 5 shows the big amount of spherical particles contained in Nc-FA. In contrast, the Si-F and R-MF are composed of angular particles.

Table 5 shows the composition of type CEM I/52.5R cement used to make the mortars. A commercial plasticizer (NEOPLAST) was mixed directly into the water at a content of 0.1 mL. This admixture is a conventional plasticizer for mortars, and it is used to improve their workability and reduce the amount of mixing water. The manufacturer recommends adding 10 mL of admixture per 50 kg of cement, which will be previously diluted in the mixing water.

### 2.3. Specimen Preparation and Testing Procedures

Two properties were tested to evaluate the fresh mortar: the consistency index and workability. The hardened mortar was characterized by studying the short- and long-term properties: dry bulk density, compressive strength, adhesive strength, water absorption by immersion, capillary water absorption, water vapor permeability and dimensional instability (shrinkage). Four mixes per mortar type were made. Table 6 shows the test standards and the climatic conditions of the three climatic chambers used in this work. The test methods are described in the correspondent standards.

### 2.4. Mortars Composition

The mixing procedure was similar to that described by Jimenez et al. [13]. Aggregates were used with their natural moisture, which was 0.41% in NA and 0.98% for FRMA. Recycled aggregates were not pre-saturated. The dry mass of each component (Table 7) was calculated as follows:

- The reference mortar (M1) was made using a constant dry mass proportion of 1:7:0.6 (CEM-I:NA:Si-F). Since the replacement of Si-F by Nc-FA was made by weight (Table 1), the rest of the mortars in Family-1 (M2 and M3) also satisfy the dry mass ratio 1:7:0.6;
- In Family-2 and Family-3, NA was replaced with FRCA by volume [16,17]. The dry mass of FRMA (in M4, M5, M6 and M8) was calculated taking into account the dry particle density of NA and FRMA using the following expressions:
  $$Dry\;mass\;of\;FRMA\;\left(g\right)=\;Dry\;mass\;of\;NA\;\left(g\right)\cdot \frac{Dry\;particle\;density\;of\;FRMA\;\left(\frac{g}{cm^{3}}\right)}{Dry\;particle\;density\;of\;NA\;\left(\frac{g}{cm^{3}}\right)}$$

## 3. Results and Discussion

### 3.1. Consistency of Fresh Mortar

The amount of water was adjusted experimentally to achieve a consistency of 175 ± 10 mm according to UNE-EN 1015-3:2000 [39]. The amount of water to maintain the consistency decreased approximately by 4% with the incorporation of Nc-FA in Family-1 and Family-2 (Table 7). This is justified by two reasons: (i) the spherical shape of Nc-FA particles acting as lubricants, in opposition to the Si-F particles, which have more fracture faces hindering the flow of the mortar (Figure 5); and (ii) the greater filler effect of the Nc-FA as a result of a more continuous particle size distribution (Figure 3) that fills the voids between sand aggregates better, therefore generating fewer free pores that are available for water. Khatib [48] showed that viscosity improves thanks to the incorporation of FA, while the amount of required superplasticizer decreases. Bilir et al. [49] concluded that the total replacement of sand with Nc-FA decreased the consistency of the mix by 13.4%, but in all cases, the workability was maintained between 198 mm (0% substitution) and 168 mm (100% substitution).

The use of FRMA increased the (w/c) ratio by 13.2% (M1 vs. M4), 16.2% (M2 vs. M5) and 17.2% (M3 vs. M6). These results agree with those presented by Ledesma et al. [16], who found increases of 17.6% in an M-10 mortar made with 50% of natural sand and 50% of recycled aggregates from masonry waste.

The use of R-MF (family-3) increased the (w/c) ratio by 7.4% (M1 vs. M7) and 6.3% (M4 vs. M8). This is due to the higher water absorption of the powdered recycled aggregates and to having used recycled aggregate [21,22] not previously saturated.

### 3.2. Workability of Fresh Mortar

Table 8 shows the workability of fresh mortar and the effect of the incorporation of Nc-FA, FRMA and RMF expressed as the percentage of variation.

The substitution of Si-F with Nc-FA improves the workability of the mortar by 11.04% in Family-1 and by 17.95% in Family-2. As with consistency, this may be due to the spherical shape of the Nc-FA particles (Figure 5). The mortars made with FRMA have a significant reduction in their workability time: M4 showed a value of −52.15% relative to M1, M5 −53.07% compared to M2, M6 −49.17% compared to M3 and, finally, M8 −50.97% compared to M7. Ledesma et al. [16] showed a loss of workability of 25% in those cases when the percentage of substitution of natural sand for recycled sand is 50%; on the other hand, Jimenez et al. [13] showed a loss of 55% when the percentage of substitution was 40%, consequently these latter values are more similar to those found in the mortars of our study.

The replacement of Si-F with R-MF shows that the workability decreases. If one compares M7 with M1, a decrease of 4.91% is observed, and for M4 and M8, there is a decrease of 2.56%. The above results are explained by the higher water absorption of recycled materials and their use in the dry state without prior pre-saturation [13,15,16,17].

### 3.3. Bulk Density of Hardened Mortar

Table 9 shows the bulk density of hardened mortar at 28 days. Family-1 and Family-2 show that the substitution of Si-F with Nc-AF increases the density of the mortar, which is due to the higher bulk density of Nc-FA (Table 4) and its ability to fill voids in the mortar.

The replacement of NA with FRMA decreases the bulk density by approximately 6% (M4 vs. M1; M5 vs. M2; M6 vs. M3; M8 vs. M7), which is justified by the lower density of FMRA particles compared to NA particles.

When Si-F is replaced with R-MF (M7 vs. M1 and M8 vs. M4), the bulk density decreases approximately by 3%.

### 3.4. Compressive Strength of Hardened Mortar

**Figure 6:**
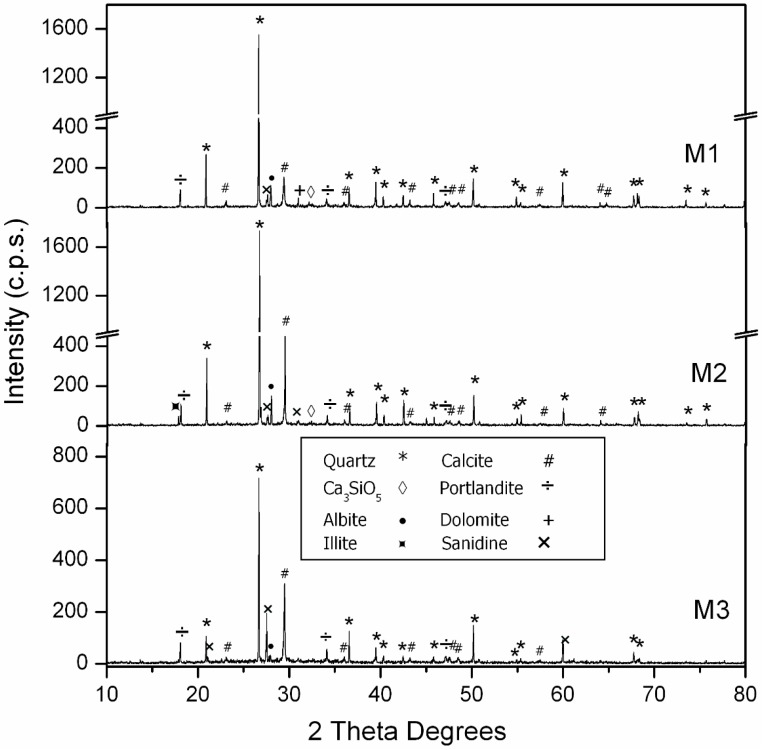
Powder X-ray diffraction (PRXD) diagrams of Family-1’s mortars.

**Figure 7:**
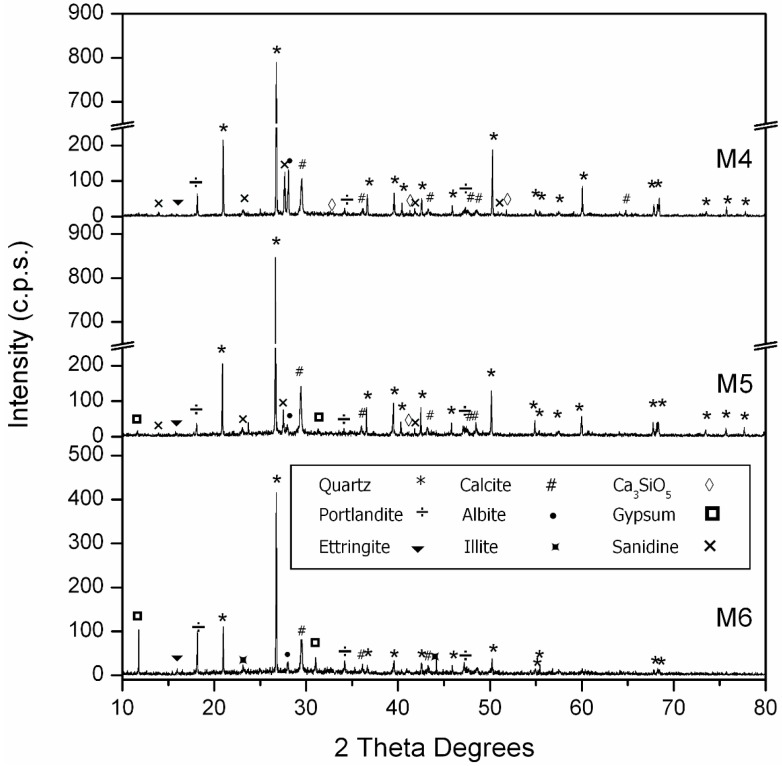
Powder X-ray diffraction (PRXD) diagrams of Family-2’s mortars.

Table 10 shows the compressive strength at four ages: 7, 28, 90 and 180 days. Analyzing the data at 28 days, the substitution of Si-F with Nc-FA increases the compressive strength by more than 12% in Family-1 (M3 vs. M1) and by more than 10% in Family-2 (M6 vs. M4). This increased resistance at 28 days can be explained, firstly, by the higher density of mortars that incorporate Nc-FA and the lower w/c ratio required to achieve the consistency of reference and, secondly, by the FA pozzolanic reaction with Ca(OH)_2_ produced by the cement hydration. Figure 6 and Figure 7 show that after 90 days, the major phase is quartz (33-1161) [35], followed by calcite (05-0586) [35]. The amount of calcite grows in comparison with other minor phases by increasing the amount of Nc-FA used as filler. When increasing the percentage of Nc-FA, an increase of portlandite (04-0733) [35] is also observed. Minor phases are related to the aggregates. In Family-1, the presence of dolomite (36-0426) [35] is due to the NA aggregate (Figure 2), and in Family-2, the presence of gypsum (42-0551) [35] is due to the FRMA aggregate (Figure 2). In Family-2, a small amount of ettringite is detected. The remaining minor phases are identical in both families: Ca_3_SiO_5_ (42-0551) [35], albite (10-0393) [35], illite (02-0056) [35] and sanidine (10-0357) [35]. The positive effect of the incorporation of FA grows as time goes by. In Family-1, the use of Nc-FA increases the compressive strength by 15.7% (M3 vs. M1) and by 17.6 % (M3 vs. M1) at 90 and 180 days, respectively. This trend is confirmed in Family-2, although the increases of resistance are lower, 6.8% (M6 vs. M4) and 8.1% (M6 vs. M4) at 90 and 180 days, respectively. Therefore, using Nc-FA can be beneficial insofar as it allows saving cement and producing an environmentally more sustainable mortar.

Bilir et al. [49] studied the effect of FA as fine aggregate in mortars. These authors showed that a replacement ratio of 30% of FA with natural sand entailed an increase of compressive strength. When FA was replaced with sand at a ratio of 100%, compressive strength decreased by 73.1% at 28 days. Fanghui et al. [50] made mortar by replacing cement with FA at levels of 20% and 40% by mass. An increase of fly ash content decreased the compressive strength of mortar at early ages. However, at later ages, the compressive strength was higher than that of the mortar made only with cement.

The substitution of NA for FRMA decreases the compressive strength by between 10.8% and 14.8% at 28 days (M4 vs. M1; M5 vs. M2; M6 vs. M3; M8 vs. M7), which can be explained by the lower mechanical strength of the recycled aggregates and the higher w/c ratio. Ledesma et al. [16] observed that when the substitution ratio of natural sand with recycled sand is 50%, the mechanical strength decreases by 11.3%. However, the use of FRMA and Nc-FA only decreases the mechanical strength of the reference mortar (M1) by 3% at 180 days. As a result, this alternative is also feasible when considering the production of an environmentally-sustainable mortar.

The worst results in terms of mechanical strength were obtained in mortars made with FRMA and R-MF. Substituting Si-F with R-MF is not a good alternative since the mechanical strength decreases by more than 20% at 28 days, which would involve increasing the amount of cement in the manufacture of the mortar in order to ensure the mechanical strength. In addition, the pulverization of the waste would increase the energy embedded within the material, and consequently, the manufacturing of the mortar would have a more severe environmental impact.

### 3.5. Water Absorption by Immersion

**Figure 8:**
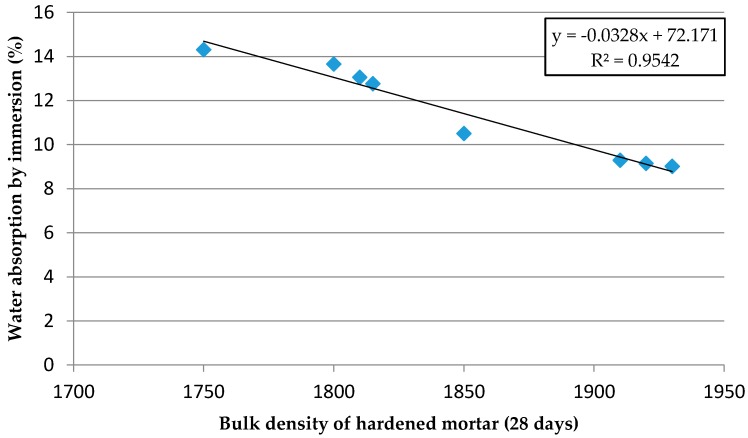
Water absorption by immersion vs. effective bulk density of hardened mortar.

The water absorption by immersion is related to the porosity of the mortar. Table 11 shows that replacement of Si-F by Nc-FA reduces the water absorption by immersion by up to 2.9% in mortars from Family-1 and by 6.5% in mortars from Family-2, which is due to Nc-FA having a greater effect of filling (Figure 3) compared to Si-FA. Figure 8 shows that water absorption by immersion is inversely proportional to the bulk density of the hardened mortar.

The use of FMRA increases the water absorption by immersion up to 47% in Family-2 (M4 vs. M1) and by 36.1% in Family-3 (M8 vs. M7), which is justified by the higher porosity of the recycled aggregates. The use of Nc-FA partially mitigates these results.

Substituting Si-F with R-MF increases water absorption by immersion by 13.1% (M1 vs. M7) in mortars made with NA and by 4.8% (M8 vs. M4) in mortars made with FRMA. The mortar made with FRMA and R-MF has the highest degree of water absorption by immersion, because of the higher porosity of the particles of the recycled aggregates.

### 3.6. Capillary Water Absorption

Unlike water absorption by immersion, Table 11 shows that the replacement of Si-F with Nc-FA slightly increases the capillary water absorption by 4.29% in the mortars from Family-1 and 6.59% in the mortars from Family-2. These results are related to the mortars’ porous microstructure. Corinaldesi [51] observed a higher capillary water absorption in mortar made with fine crushed brick aggregates (FCB) where the amount of micropores (<0.1 µm) and mesopores (0.1–1 µm) were higher than mortar made with coarse crushed brick aggregates (CCB). By contrast, mortars made with CCB showed a greater proportion of macropores (>1 µm) than mortar made with FCB.

Using FMRA increases the capillary water absorption by up to 32.88% in Family-2 (M6 vs. M3) and 27.27% in Family-3 (M8 vs. M7), which is due to the higher porosity of the recycled aggregates. The obtained values are of the same order of magnitude of those obtained by Jiménez et al. [13] and Ledesma et al. [16]. The results are coherent with those obtained by Silva et al. [52] and Corinaldesi et al. [51].

Substituting Si-F with R-MF increases the capillary water absorption by 25.71% (M1 vs. M7) in mortars made with NA and by 23.08% (M8 vs. M4) in mortars made with FRMA. The mortar made with FRMA and R-MF has the highest degree of capillary water absorption.

### 3.7. Water Vapor Permeability

Table 12 shows that the replacement of Si-F by Nc-FA decreases the water vapor permeability by up to 31.48% in mortars from Family-1 and 14.77% in mortars from Family-2. These results are justified by Nc-FA having a greater effect of filling (Figure 3) compared to Si-FA, as for water absorption by immersion. Family-2 shows lower values of permeability than those from Family-1, which can be due to the higher percentage of FRMA particles that are smaller than 0.063 mm in comparison with NA (Table 2). This in turn can explain why the percentage of variation of the water vapor permeability when using Nc-FA is lower in Family-2 than in Family-1.

Using FMRA reduces the water vapor permeability by up to 52.39% in Family-2 (M4 vs. M1) and by 31.21% in Family-3 (M8 vs. M7). These results are also justified by the higher percentage of FRMA particles that are smaller than 0.063 mm in comparison with NA (Table 2).

The substitution of Si-F with R-MF decreases the water vapor permeability by 37.63% (M1 vs. M7) in mortars made with NA and by 23.19% (M8 vs. M4) in mortars made with FRMA. The mortar made with FRMA and R-MF has the lowest value of water vapor permeability, which is justified by the filler effect that results from the combined use of FRMA and R-MF.

### 3.8. Drying Shrinkage

**Figure 9:**
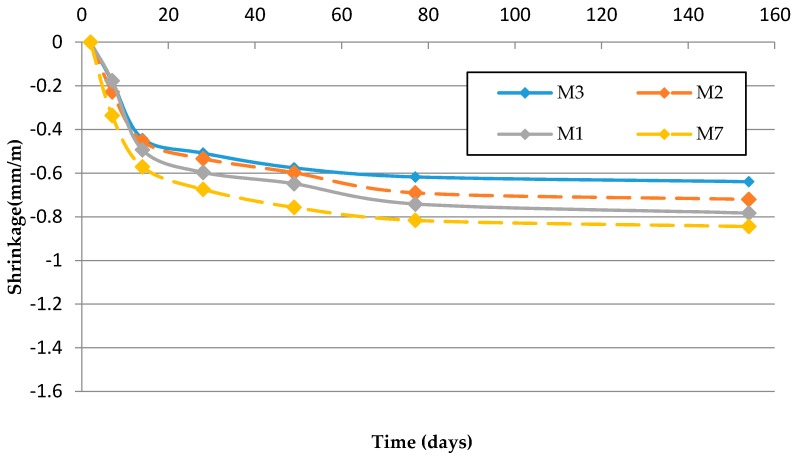
Drying shrinkage (Family-1 and Family-3).

**Figure 10:**
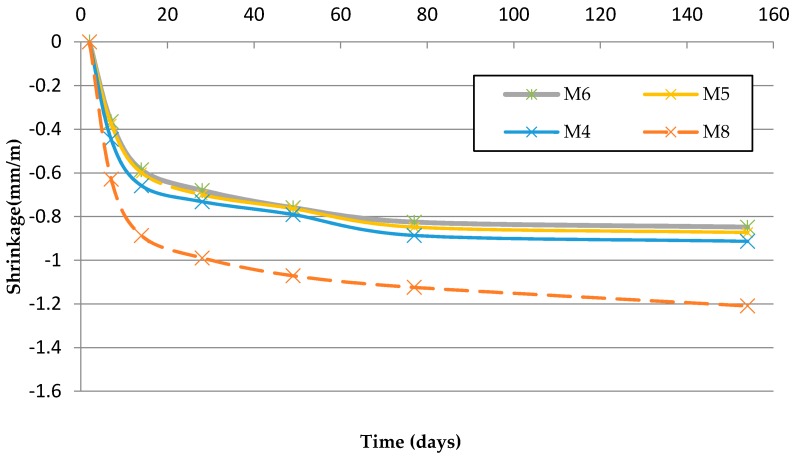
Drying shrinkage (Family-2 and Family-3).

Table 13 and Figure 9 and Figure 10 show that the replacement of Si-F with Nc-FA decreases shrinkage by up to 17.95% in mortars from Family-1 and 6.59% in mortars from Family-2. These results can be explained by the lower w/c ratio and greater mechanical strength of mortars made with Nc-FA. Most research carried out shows that the incorporation of coal bottom or fly ash in cement-based materials allows a better dimensional stability [53,54]. The porous particle structure of mortar with Nc-FA is beneficial for decreasing the drying shrinkage of the mortar.

Using FMRA increases the dry shrinkage by up to 32.81% in Family-2 (M6 vs. M3) and by 44% in Family-3 (M8 vs. M7). These results are justified by the higher w/c ratio and lower mechanical strength of mortars made with FRMA. These results coincide with those provided by Ledesma et al. [16].

The substitution of Si-F with R-MF increases the drying shrinkage 7.69% (M1 vs. M7) in mortars made with NA and by 32.97% (M8 vs. M4) in mortars made with FRMA. The mortar made with FRMA and R-MF is the one with the highest value of drying shrinkage. In addition, it is the mortar with the highest w/c ratio and the lowest mechanical strength, which can explain the results. Silva et al. [21] also highlighted the negative effect of the incorporation of ceramic powder material on the drying shrinkage of mortars.

## 4. Conclusions

The spherical shape of Nc-FA particles improves the workability of mortars and allows using less mixing water for a given consistency. The greater bulk density of Nc-FA improves the bulk density of the fresh and hardened mortar. This improves the mechanical strength at 180 days by 17.6% and by 8.1% in mortars made with siliceous sand and mixing siliceous natural sand and recycled sand from CDW, respectively. This conclusion allows assuming that it is possible to reduce the cement content in the manufacture of mortars without reducing their mechanical performance. Both the water absorption by immersion and the water vapor permeability decrease as the replacement ratio of Si-F with Nc-FA increases. However, the capillary water absorption slightly increases. Shrinkage also decreases. Therefore, the replacement of Si-F by Nc-FA is a viable alternative and allows producing more environmentally-sustainable mortars.

The combined effect of recycled sand from masonry waste and Nc-FA (M6) only decreases by 3% the mechanical strength of the reference mortar made with siliceous natural sand and siliceous filler (M1) at 180 days; hence, it is also considered a viable and environmentally-friendly alternative. However, it impacts the workability very negatively; therefore, a new study about new admixtures that can improve this property should be carried out. The water absorption by immersion and the water vapor permeability increase, but the capillary water absorption decreases compared to the reference mortar. Incorporating recycled sand from CDW increases shrinkage, although the increases may be acceptable for less than 50% of recycled sand. This alternative allows reducing the consumption of natural sand while involving the recycling of the masonry waste and the non-conforming fly ash, both of which are currently generating an environmental problem on a worldwide scale.

Substituting Si-F with R-MF is not a good alternative, since workability is negatively affected, density decreases, mechanical resistance is reduced by more than 20% at 28 days and the drying shrinkage of the mortars significantly increases. This alternative would need to increase the amount of cement to ensure the mechanical performance of the mortars, which in turn would increase CO_2_ emissions. In addition, the pulverization of recycled aggregates from CDW would increase the embedded energy of the material, and it would entail the manufacture of an environmentally-unfriendly mortar.

## Figures and Tables

**Table 1:** Experimental design: nomenclature. Si-F, siliceous filler; Nc-FA, non-conforming fly ash.

Family	Mortar Type	NA/FRMA (% in Volume)	Filler (% in Mass)		
			Si-F	Nc-FA	R-MF
Family-1	M1	100/0	100	0	0
	M2	100/0	50	50	0
	M3	100/0	0	100	0
Family-2	M4	50/50	100	0	0
	M5	50/50	50	50	0
	M6	50/50	0	100	0
Family-3	M7	100/0	0	0	100
	M8	50/50	0	0	100

**Table 2:** Particle size distribution.

Sieve Size (mm)	Material Passing (%)				
	NA	FRMA	Si-F	Nc-FA	R-MF
4	100.00	100.00	--	--	--
2	87.00	85.00	--	--	--
1	73.00	59.00	--	--	100.00
0.5	53.00	39.00	--	100.00	98.65
0.25	23.00	21.00	--	96.63	77.50
0.125	8.00	12.00	100.00	90.36	76.76
0.063	3.20	9.00	87.33	82.01	75.22
Cu	4	10	--	--	--

Cu: uniformity coefficient.

**Table 3:** Characterization of NA and FRMA.

Characteristic	Standard	NA	FRMA	Limit Set by UNE-EN 13139:2003 [26]
Fines content (%) ^(a)^	UNE-EN 933-1:2012 [25]	3.2	9.0	≤8
Sand equivalent (%)	UNE-EN 933-8:2012 [32]	83	86	No limit
Dry sample density ^(b)^ ρ_rd_ (g/cm^3^)	UNE-EN 1097-6:2014 [33]	2.63	2.14	No limit
Water absorption ^(b)^ (%)	UNE-EN 1097-6:2014 [33]	0.79	9.00	No limit
Friability coefficient (%)	UNE 83115:1989 [34]	15	32	No limit
Acid soluble sulfates (% SO_3_)	UNE-EN 1744-1:2010 [30]	<0.01	1.04	≤0.8
Total sulfurs (% SO_3_)	UNE-EN 1744-1:2010 [30]	<0.01	1.04	≤1
Water soluble chlorides (% Cl^−^)	UNE-EN 1744-1:2010 [30]	<0.01	<0.01	≤0.15
Soluble salts 1:2 (%)	Gravimetric	0.128	1.159	≤1

^(a)^ Finer than 0.063 mm; ^(b)^ fraction 0.063/4 mm.

**Table 4:** Characterization of Si-F, Nc-FA and R-MF.

Characteristic	Standard	Si-F	Nc-FA	R-MF	Limit set by UNE-EN 13139:2003 [26]
% SO_3_	UNE-EN 196-2 [36]	0.2001	0.1220	1.3797	≤0.8
% Cl^−^	UNE-EN 196-2 [36]	0.0144	0.0462	0.0319	≤0.15
Bulk density (g/cm^3^)	NLT 176:1992 [37]	0.6890	0.9100	0.8340	---
Particle density (g/cm^3^)	UNE 80103:1986 [38]	2.4190	1.9400	2.3430	---

**Table 5:** Characteristics of CEM I/52.5R.

**Constituents**	
Clinker (%)	97
Minority (%)	3
**Chemical Characteristics**	
SiO_2_ (%)	20.16
CaO (%)	61.58
Al_2_O_3_ (%)	4.52
Fe_2_O_3_ (%)	2.59
MgO (%)	0.98
SO_3_ (%)	3.46
K_2_O (%)	1.00
LOI (%)	2.83
**Physical Characteristics**	
Density (g/cm^3^)	3.11
Blaine specific area (cm^2^/g)	4619
Expansion (Le Châtelier) (mm)	≤10
Initial set (min)	≥45
Final set (min)	≤720
**Mechanical Characteristics**	
Compression strength at 7 days (MPa)	≥30
Compression strength at 28 days (MPa)	≥52.5

**Table 6:** Mortar characterization test.

**Properties of Fresh Mortar**	**Standard**	**Specimens and Dimensions**	**Climatic Chamber**	**Curing Time**
Consistency	UNE-EN 1015-3:2000 [39]	--	--	--
Workability	UNE-EN 1015-9:2000 [40]	4	--	--
**Properties of Hardened Mortar**	**Standard**	**Specimens and Dimensions**	**Climatic Chamber**	**Curing Time**
Dry bulk density	UNE-EN 1015-10:2000 [41]	4 Prismatic 40 mm × 40 mm × 160 mm	Chamber-1 (7 days) Chamber-2 (rest of curing time)	28 days
Compressive strength	UNE-EN 1015-11:2000 [42]	8 Prismatic 40 mm × 40 mm × 80 mm	Chamber-1 (7 days) Chamber-2 (rest of curing time)	7, 28, 90, 180 days
Adhesive strength	UNE-EN 1015-12:2000 [43]	4 Circular 50 mm diameter, 10 mm-thick	Chamber-1 (7 days) Chamber-2 (rest of curing time)	28 days
Water absorption by immersion	UNE-EN 83980-2014 [44]	12 Prismatic 40 mm × 40 mm × 80 mm	Chamber-1 (7 days) Chamber-2 (rest of curing time)	28 days
Water absorption by capillarity	UNE-EN 1015-18:2003 [45]	12 Prismatic 40 mm × 40 mm × 80 mm	Chamber-1 (7 days) Chamber-2 (rest of curing time)	28 days
Water vapor permeability	UNE-EN 1015-19:1999 [46]	4 Circular 160 mm (0.02 m^2^ surface) diameter, 15 mm-thick	Chamber-1 (2 days) Chamber-3 (rest of curing time)	28 days
Shrinkage	UNE 83831:2010 EX [47]	6 Prismatic 40 mm × 40 mm × 160 mm	Chamber-1 (7 days) Chamber-2 (rest of curing time)	Up to 154 days

Climatic conditions: Chamber-1: Tª = 20 ± 2 °C RH = 95% ± 5%; Chamber-2: Tª = 20 ± 2 °C RH = 65% ± 5%; Chamber-3: Tª = 20 ± 2 °C RH = 50% ± 5%.

**Table 7:** Mortars’ composition.

Family	Mortar Type	Mix Proportions–Dry Mass									
		NA (g)	FRMA (g)	Si-F (g)	Nc-FA (g)	R-MF (g)	CEM-I (g)	Water (g)	Admixture (cm^3^)	Consistency (mm)	w/c Total
Family-1	M1	3500	0	300	0	0	500	605	0.1	176	1.21
	M2	3500	0	150	150	0	500	587	0.1	177	1.17
	M3	3500	0	0	300	0	500	579	0.1	172	1.16
Family-2	M4	1750	1424	300	0	0	500	709	0.1	176	1.42
	M5	1750	1424	150	150	0	500	683	0.1	174	1.37
	M6	1750	1424	0	300	0	500	681	0.1	173	1.36
Family-3	M7	3500	0	0	0	300	500	648	0.1	179	1.30
	M8	1750	1424	0	0	300	500	754	0.1	181	1.51

**Table 8:** Workability of fresh mortar.

Family	Mortar	Mean (min)	SD	Δ(%)Nc-FA	Δ(%)FRMA	Δ(%)R-MF
Family-1	M1	163	11	0.00	0.00	0.00
	M2	179	15	9.82	0.00	--
	M3	181	7	11.04	0.00	--
Family-2	M4	78	4	0.00	−52.15	0.00
	M5	84	5	7.69	−53.07	--
	M6	92	5	17.95	−49.17	--
Family-3	M7	155	4	--	0.00	−4.91
	M8	76	5	--	−50.97	−2.56

**Table 9:** Bulk density of hardened mortar at 28 days.

Family	Mortar	Mean (kg/m^3^)	SD	Δ(%)Nc-FA	Δ(%)FRMA	Δ(%)R-MF
Family-1	M1	1910	8	0.00	0.00	0.00
	M2	1920	15	0.52	0.00	--
	M3	1930	13	1.05	0.00	--
Family-2	M4	1800	11	0.00	−5.76	0.00
	M5	1810	10	0.56	−5.73	--
	M6	1815	7	0.83	−5.96	--
Family-3	M7	1850	12	--	0.00	−3.14
	M8	1750	11	--	−5.41	−2.78

**Table 10:** Compressive strength of hardened mortar.

Family	Mortar	7 Days		28 Days					90 Days		180 Days	
		Mean (MPa)	SD	Mean (MPa)	SD	Δ(%)Nc-FA	Δ(%)FRMA	Δ(%)R-MF	Mean (MPa)	SD	Mean (MPa)	SD
Family-1	M1	14.28	0.23	15.76	0.96	0.00	0.00	0.00	16.34	0.71	16.48	0.75
	M2	14.84	0.35	16.37	0.56	3.87	0.00	--	17.88	0.83	17.97	1.01
	M3	15.57	0.53	17.74	0.5	12.56	0.00	--	18.91	0.84	19.38	1.02
Family-2	M4	12.85	0.88	13.68	0.72	0.00	−13.20	0.00	14.53	0.65	14.76	0.63
	M5	13.20	1.14	14.2	1.59	3.80	−13.26	--	15.05	1.06	15.32	1.80
	M6	14.10	0.63	15.11	0.81	10.45	−14.83	--	15.52	0.49	15.96	0.89
Family-3	M7	11.32	0.51	11.98	0.58	--	0.00	−23.98	13.2	0.48	12.94	0.43
	M8	9.88	0.37	10.68	0.38	--	−10.85	−21.93	11.23	1.1	10.82	0.87

**Table 11:** Water absorption by immersion and capillary action of hardened mortar.

Family	Mortar	Absorption by Immersion					Capillary Water Absorption 90 min				
		Mean (%)	SD	Δ(%)Nc-FA	Δ(%)FRMA	Δ(%)R-MF	Mean (kg/(m^2^·min^−0.5^)	SD	Δ(%)Nc-FA	Δ(%)FRMA	Δ(%)R-MF
Family-1	M1	9.29	0.05	0.00	0.00	0.00	0.7	0.02	0.00	0.00	0.00
	M2	9.15	0.07	−1.51	0.00	--	0.72	0.06	2.86	0.00	--
	M3	9.02	0.13	−2.91	0.00	--	0.73	0.03	4.29	0.00	--
Family-2	M4	13.66	0.23	0.00	47.04	0.00	0.91	0.03	0.00	30.00	0.00
	M5	13.06	0.07	−4.39	42.73	--	0.94	0.04	3.30	30.56	--
	M6	12.77	0.06	−6.52	41.57	--	0.97	0.08	6.59	32.88	--
Family-3	M7	10.51	0.14	--	0.00	13.13	0.88	0.05	--	0.00	25.71
	M8	14.31	0.14	--	36.16	4.76	1.12	0.07	--	27.27	23.08

**Table 12:** Water vapor permeability of hardened mortar.

Family	Mortar	Mean	SD	Δ(%)Nc-FA	Δ(%)FRMA	Δ(%)R-MF
Family-1	M1	13.82	0.24	0.00	0.00	0.00
	M2	10.01	0.13	−27.57	0.00	--
	M3	9.47	0.28	−31.48	0.00	--
Family-2	M4	7.72	0.32	0.00	−44.14	0.00
	M5	6.78	0.18	−12.18	−50.94	--
	M6	6.58	0.2	−14.77	−52.39	--
Family-3	M7	8.62	0.21	--	0.00	−37.63
	M8	5.93	0.15	--	−31.21	−23.19

**Table 13:** Shrinkage at 154 days.

Family	Mortar	Mean (mm/m)	SD (mm/m)	Δ(%)Nc-FA	Δ(%)FRMA	Δ(%)R-MF
Family-1	M1	−0.78	0.14	0.00	0.00	--
	M2	−0.72	0.16	−7.69	0.00	--
	M3	−0.64	0.15	−17.95	0.00	--
Family-2	M4	−0.91	0.17	0.00	16.67	--
	M5	−0.87	0.13	−4.40	20.83	--
	M6	−0.85	0.20	−6.59	32.81	--
Family-3	M7	−0.84	0.16	--	0.00	7.69
	M8	−1.21	0.14	--	44.00	32.97

## References

[1] Eurostat Statistics Explained. Energy Production and Imports. http://ec.europa.eu/eurostat/statistics-explained/index.php/Energy_production_and_imports.

[2] European Coal Combustion Products Association e. V. (ECOBA). http://www.ecoba.com/ecobaccpprod.html.

[3] Bech N., Feuerborn H.J. Ash quality in Europe-Primary and secondary measures. Proceedings of the World of Coal Ash Conference.

[4] Carroll R.A. Coal Combustion Products in the United Kingdom and the Potential of Stockpile Ash. Proceedings of the Word of Coal Ash Conference.

[5] European Union Decision 2014/955/UE. http://eur-lex.europa.eu/legal-content/ES/TXT/?uri=celex%3A32014D0955.

[6] (2008). Directive 2008/98/EC of the European Parliament and of the Council of 10 November 2008 on Waste. Official Journal of the European Union.

[7] European Chemical Agency (ECHA). http://echa.europa.eu/es/regulations/reach.

[8] UNE-EN 450-1 (2013). Fly Ash for Concrete—Part 1: Definition, Specifications and Conformity Criteria (in Spanish).

[9] UNE-EN 450-2 (2006). Fly Ash for Concrete—Part 2: Conformity Evaluation.

[10] Ahmaruzzaman M. (2010). A review on the utilization of fly ash. Prog. Energ. Combust..

[11] UNE-EN 933-10 (2010). Tests for Geometrical Properties of Aggregates—Part 10: Assessment of Fines—Grading of Filler Aggregates (Air Jet Sieving).

[12] Saviour M.N. (2012). Environmental impact of soil and sand mining: A review. Int. J. Sci. Environ. Technol..

[13] Jiménez J.R., Ayuso J., López M., Fernández J.M., de Brito J. (2013). Use of fine recycled aggregates from ceramic waste in masonry mortar manufacturing. Constr. Build. Mater..

[14] Neno C., de Brito J., Veiga R. (2014). Using fine recycled concrete aggregate for mortar production. Mater. Res..

[15] Ledesma E.F., Jiménez J.R., Fernández J.M., Galvín A.P., Agrela F., Barbudo A. (2014). Properties of masonry mortars manufactured with fine recycled concrete aggregates. Constr. Build. Mater..

[16] Ledesma E.F., Jiménez J.R., Ayuso J., Fernández J.M., de Brito J. (2015). Maximum feasible use of recycled sand from construction and demolition waste for eco-mortar production—Part-I: Ceramic masonry waste. J. Clean. Prod..

[17] Fernández-Ledesma E., Jiménez J.R., Ayuso J., Corinaldesi V., Iglesias-Godino F.J. (2016). A proposal for the maximum use of recycled concrete sand in masonry mortar design. Mater. Constr..

[18] European Commission (DG ENV) (2011). Management of CDW. http://ec.europa.eu/environment/waste/pdf/2011_CDW_Report.pdf.

[19] Kim K., Shin M., Cha S. (2013). Combined effects of recycled aggregate and fly ash towards concrete sustainability. Constr. Build. Mater..

[20] Kou S.C., Poon C.S. (2013). Long-term mechanical and durability properties of recycled aggregate concrete prepared with the incorporation of fly ash. Cem. Concr. Compos..

[21] Silva J., de Brito J., Veiga R. (2009). Incorporation of fine ceramics in mortars. Constr. Build. Mater..

[22] Braga M., de Brito J., Veiga R. (2012). Incorporation of fine concrete aggregates in mortars. Constr. Build. Mater..

[23] UNE-EN 933-11 (2009). Tests for Geometrical Properties of Aggregates—Part 11: Classification Test for the Constituents of Coarse Recycled Aggregate.

[24] UNE-EN 1097-2 (2010). Tests for Mechanical and Physical Properties of Aggregates—Part 2: Methods for the Determination of Resistance to Fragmentation.

[25] UNE-EN 933-1 (2012). Tests for Geometrical Properties of Aggregates—Part 1: Determination of Particle Size Distribution—Sieving Method.

[26] UNE-EN 13139 (2003). Aggregates for Mortar.

[27] Jiménez J.R., Ayuso J., Agrela F., López M., Galvín A.P. (2012). Utilisation of unbound recycled aggregates from selected CDW in unpaved rural roads. Res. Conser. Recycl..

[28] Jiménez J.R., Ayuso J., Galvín A.P., López M., Agrela F. (2012). Use of mixed recycled aggregates with a low embodied energy from non-selected CDW in unpaved rural roads. Constr. Build. Mater..

[29] Tovar-Rodríguez G., Barra M., Pialarissi S., Aponte D., Vázquez E. (2013). Expansion of mortars with gypsum contaminated fine recycled aggregates. Constr. Build. Mater..

[30] UNE-EN 1744-1 (2010). Tests for Chemical Properties of Aggregates. Part 1: Chemical Analysis.

[31] UNE 146507-1 (1999). Test for Aggregates. Determination of the Potential Reactivity of Aggregates. Chemical Method. Part 1: Determination of the Reactivity Alkali-Silica and Alkali-Silicate.

[32] UNE-EN 933-8 (2012). Tests for Geometrical Properties of Aggregates. Part 8: Assessment of Fines. Sand Equivalent Test.

[33] UNE-EN 1097-6 (2014). Teste for Mechanical and Physical Properties of Aggregates—Part 6: Determination of Particle Density and Water Absorption.

[34] UNE 83115 (1989). Aggregates for Concrete. Determination of the Coefficient of Friability of the Sands.

[35] International Center for Diffraction Data, Inorganic Phases (1995). Power Diffraction File.

[36] UNE-EN 196-2 (2014). Methods of Testing Cement—Part 2: Chemical Analysis of Cement.

[37] NLT 176 (1992). Bulk Density of Mineral Powder in Toluene.

[38] UNE 80103 (2013). Test Methods of Cements. Physical Analysis. Density determination by the Standard le Chatelier Flask.

[39] UNE-EN 1015-3 (2000). Methods of Test for Mortar for Masonry. Part 3: Determination of Consistence of Fresh Mortar (by Flow Table).

[40] UNE-EN 1015-9 (2000). Methods of Test for Mortar for Masonry—Part 9: Determination of Workable Life and Correction Time of Fresh Mortar.

[41] UNE-EN 1015-10 (2000). Methods of Test for Mortar for Masonry—Part 10: Determination of Dry Bulk Density of Hardened Mortar.

[42] UNE-EN 1015-11 (2000). Methods of Test for Mortar for Masonry—Part 11: Determination of Flexural and Compressive Strength of Hardened Mortar.

[43] UNE-EN 1015-12 (2000). Methods of Test for Mortar for Masonry—Part 12: Determination of Adhesive Strength of Hardened Rendering and Plastering Mortars on Substrates.

[44] UNE 83980 (2014). Concrete Durability. Test Methods. Determination of the Water Absorption, Density and Accessible Porosity for Water in Concrete.

[45] UNE-EN 1015-18 (2003). Methods of Test for Mortar for Masonry—Part 18: Determination of Water Absorption Coefficient due to capillary action of hardened mortar.

[46] UNE-EN 1015-19 (1999). Methods of Test for Mortar for Masonry—Part 19: Determination of Water Vapour Permeability of Hardened Rendering and Plastering Mortars.

[47] UNE 83831 (2010). Methods of Test for Hardened Mortar for Masonry—Determination of Dimensional Stability of Hardened Mortar for Masonry.

[48] Khatib J.M. (2008). Performance of self-compacting concrete containing fly ash. Constr. Build. Mater..

[49] Bilir T., Gencel O., Topcu I.B. (2015). Properties of mortars with fly ash as fine aggregate. Constr. Build. Mater..

[50] Fanghui H., Qiang W., Jingjing F. (2015). The differences among the roles of ground fly ash in the paste, mortar and concrete. Constr. Build. Mater..

[51] Corinaldesi V. (2012). Environmentally-friendly bedding mortars for repair of historical buildings. Constr. Build. Mater..

[52] Silva J., de Brito J., Veiga R. (2010). Recycled red-clay ceramic construction and demolition waste for mortars production. J. Mater. Civil Eng..

[53] Rafieizonooz M., Mirza J., Salim M.R., Hussin M.W., Khankhaje E. (2016). Investigation of coal bottom ash and fly ash in concrete as replacement for sand and cement. Constr. Build. Mater..

[54] Singh M., Siddique R. (2014). Strength properties and micro-structural properties of concrete containing coal bottom ash as partial replacement of fine aggregate. Constr. Build. Mater..

